# The Diagnostic, Prognostic, and Therapeutic Utility of Molecular Testing in a Patient with Waldenstrom’s Macroglobulinemia

**DOI:** 10.3390/ijms18102038

**Published:** 2017-09-22

**Authors:** Collin K. Chin, Connull Leslie, Carolyn S. Grove, Chris Van Vliet, Chan Yoon Cheah

**Affiliations:** 1 Department of Haematology, Sir Charles Gairdner Hospital and Pathwest Laboratory Medicine WA, Nedlands 6009, Australia; Collin.Chin@health.wa.gov.au (C.K.C.); Carolyn.Grove@health.wa.gov.au (C.S.G.); 2 Department of Anatomical Pathology, Pathwest Laboratory Medicine WA, Nedlands 6009, Australia; Connull.Leslie@health.wa.gov.au (C.L.); Benjamin.VanVliet@health.wa.gov.au (C.V.V.); 3 Medical School, University of Western Australia, Crawley 6009, Australia

**Keywords:** Waldenstrom’s macroglobulinemia, *MYD88*, Bruton’s tyrosine kinase (BTK) inhibitors, molecular diagnostics

## Abstract

The application of molecular genomics and our understanding of its clinical implications in the diagnosis, prognostication and treatment of lymphoproliferative disorders has rapidly evolved over the past few years. Of particular importance are indolent B-cell malignancies where tumour cell survival and proliferation are commonly driven by mutations involving the B-cell receptor and downstream signalling pathways. In addition, the increasing number of novel therapies and targeted agents have provided clinicians with new therapeutic options with the aim of exploiting such mutations. In this case report, we highlight one such success story involving the diagnostic impact of the *MYD88*^L265P^ mutation in Waldenstrom’s macroglobulinemia (WM), its prognostic implications and effect on choice of therapy in the era of novel therapies.

## 1. Case Presentation

An 89-year-old retired office worker with comorbidities including congestive cardiac failure and atrial fibrillation (on rivaroxaban) presented with a suprapubic mass and widespread lymphadenopathy. Further history revealed unintentional weight loss of 10 kg over six months and lethargy associated with a normocytic anaemia of 92 g/L and IgM kappa paraproteinemia of 31 g/L. Positron-emission tomography computerized tomography (PET-CT) revealed extensive fluorodeoxyglucose (FDG) avid lymphadenopathy, bilateral pleural effusions, and diffuse activity in the bone marrow without evidence of high grade disease (maximum standardized uptake value [SUV_max_] 2.4). Diagnostic thoracocentesis was negative for malignancy.

**Figure 1:**
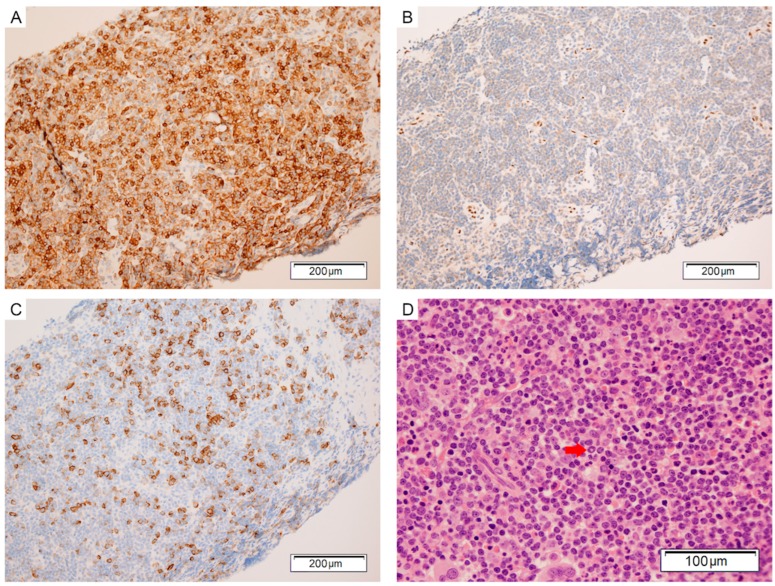
(**A**–**C**) Inguinal lymph node core biopsy; (**A**) CD10 is positive in the lymphoma cells; (**B**) LMO2 is negative in the lymphoma cells; (**C**) CD138 is positive in mature plasma cells; (**D**) Bone marrow trephine containing a diffuse infiltrate of small mature lymphocytes with interspersed plasma cells. Note the Dutcher body (arrow).

Core biopsy of an inguinal lymph node showed diffuse replacement of normal nodal architecture by small lymphocytes with interspersed plasmacytoid cells and mature plasma cells without follicular architecture or sheet-like large cell component. By immunohistochemistry, the lymphocytes were positive for cluster of differentiation 20 (CD20), B-cell lymphoma 2 (BCL2), CD10, CD23 (minor subset), Immunoglobulin D (IgD), Immunoglobulin M (IgM), kappa light chains and negative for CD3, CD5, B-cell lymphoma 6 (BCL6), Lim domain only 2 (LMO2), cyclin D1, SRY-Box 11 (SOX11), Epstein-Barr encoding region (EBER), and lambda light chains (Figure 1A,B). CD138 and Multiple Myeloma Oncogene 1 (MUM1) highlight a subpopulation of mature plasma cells (Figure 1C). The Ki-67 proliferation index was <10%. A bone marrow biopsy was hypercellular with heavy diffuse and interstitial infiltrate of small lymphocytes with similar morphology and immunophenotype, with some preservation of background trilineage haematopoiesis (Figure 1D). Both the lymphocytes and plasma cells were CD79a positive and occupied about 30% of the marrow space. Given the lack of follicularity or large cell component, the differential diagnoses favoured nodal marginal zone lymphoma or lymphoplasmacytic lymphoma although positivity for CD10 raised the possibility of diffuse patterned follicular lymphoma.

Testing for *MYD88*^L265P^ mutation was performed on formalin fixed paraffin embedded tissue by digital polymerase chain reaction (dPCR) from the lymph node biopsy and bone marrow trephine, which demonstrated the *MYD88*^L265P^ mutation present at an allele frequency of 46% and 0.69%, respectively. Confirmatory testing for *MYD88* and *CXCR4* mutation was performed on bone marrow aspirate using bi-directional Sanger sequencing on exon 5 of *MYD88* and C1013G/*CXCR4* mutation on chromosome 2q21, respectively. *MYD88*^L265P^ and *CXCR4*^WT^ (WT = wild type) were subsequently confirmed. The final diagnosis was lymphoplasmacytic lymphoma (LPL) with aberrant CD10 expression, *MYD88*^L265P^ mutation and *CXCR4*^WT^. Taken in conjunction with the IgM kappa serum paraprotein, the findings were consistent with Waldenstrom’s macroglobulinemia (WM).

In this case detection of the *MYD88*^L265P^ mutation by digital PCR in conjunction with clinical and morphologic features confirmed the diagnosis of WM despite aberrant CD10+ expression on immunohistochemistry. Although lacking symptoms of hyperviscosity, peripheral neuropathy, or other paraneoplastic sequelae, the patient’s symptomatic anaemia provided reason to commence therapy. Given the patient’s age, comorbidities, and genomic profile, he was offered enrolment in a clinical trial with a second generation BTK inhibitor with careful clinical monitoring due to his atrial fibrillation and rivaroxaban. Treatment has been well tolerated and initial response assessment is ongoing.

## 2. Discussion

### 2.1. Diagnostic Relevance of MYD88 L265P

MYD88 is an adaptor protein that mediates toll and interleukin (IL)-1 receptor signaling. *MYD88*^L265P^ is a gain of function mutation that drives lymphomagenesis by promoting cell survival through nuclear factor-κB (NF-κB). The mutation allows spontaneous assembly of a protein complex containing interleukin-1 receptor-associated kinases (IRAK1 and IRAK4), leading to increased IRAK kinase activity and downstream NF-κB activation [1]. Additionally, *MYD88*^L265P^ enhances Bruton’s tyrosine kinase (BTK) phosphorylation resulting in the downstream activation of NF-κB independent of IRAK/IRAK4 signaling [2]. *MYD88*^L265P^ is a recurring somatic mutation in WM and LPL and occurs in approximately 90% of cases [3]. *MYD88*^L265P^ can also be detected by highly sensitive assays in IgM MGUS (monoclonal gammopathy of undetermined significance) and WM patients undergoing treatment [4]. PCR is the preferred method for detecting the *MYD88*^L265P^ mutation due to its high sensitivity, rapidity, low cost, and ability to detect point mutations as compared to fluorescence in situ hybridization (FISH) studies which have a limit of resolution of 50 kilobases. Sanger sequencing for non-L265P *MYD88* mutations has been used for patients who are negative for *MYD88*^L265P^ by allele-specific polymerase chain reaction (AS-PCR) as other types of *MYD88* mutations have also been demonstrated in LPL, with a recent prospective study identifying 2 out of 57 patients with *MYD88* mutations harbouring a non-L265P mutation [5]. Single nucleotide polymorphism-based array (SNPa) may also be used to detect non-L265P mutations. A French study involving 31 patients with WM identified copy number alterations or loss of heterozygosity in 64%, although the role of these genetic aberrations in the diagnosis and treatment of WM still remains unclear [6]. Sanger sequencing remains the gold standard and is more accessible, more affordable, and quicker to perform.

*MYD88* mutation testing is of most value in diagnosing small B cell lymphomas with plasmacytic differentiation in which LPL is a strong possibility, as a positive result is highly suggestive of LPL [7]. It is important to recognise that the *MYD88*^L265P^ mutation is not specific as it is found in a small proportion of splenic marginal zone lymphomas (~15%) [8,9], and rarely in other small B cell lymphomas [7,9,10]. It is uncertain whether this represents secondary acquisition of the *MYD88* mutation or if this is an unusual primary driving event in these tumours [7]. In addition, *MYD88*^L265P^ mutation is the most frequent genomic abnormality in diffuse large B cell lymphoma (DLBCL) activated B-cell-like (ABC) subtype, as it is detected in 40% of cases [11].

### 2.2. Prognostic Implications of MYD88 in WM

In the era of novel agents, Treon et al. have reported three distinct genomic groups of WM differentiated by *MYD88* and *CXCR4* mutation status. These groups showed significantly different clinical manifestations and overall survival (OS) [12]. *MYD88*^WT^ status was shown to have lower BM disease burden but significantly inferior overall survival (4.7 years vs. >10 years; *p* = 0.0018). In contrast, *MYD88*^L265P^ *CXCR4*^WHIM/NS^ (WHIM = syndrome associated with warts, hypogammaglobulinaemia, infections, and myelokathexis; NS = nonsense mutation) was associated with significantly higher BM involvement, higher IgM paraproteinaemia and symptomatic disease, including hyperviscosity (*p* = 0.03). *MYD*^L265P^ *CXCR4*^WHIM/FS^ (FS = frameshift mutation) and *MYD*^L265P^ *CXCR4*^WT^ were associated with intermediate disease burden. The dependence of WM tumour cells on activation of NF-κB by the BTK and IRAK pathways has been demonstrated by the significant activity seen in pre-clinical and clinical studies using IRAK1/4, BTK, and NF-κB inhibitors [13,14,15,16,17,18]. Although BTK inhibition has been found to be highly effective in the treatment of WM, the efficacy is influenced by *MYD88* mutation status [5]. In patients with mutated *MYD88* but *CXCR4*^WT^, the overall response rate (ORR) to the BTK inhibitor ibrutinib is 100% with a 91.7% major response rate. Patients with wild-type *MYD88* have significantly inferior outcomes with an ORR of 60% (*p* = 0.005) and no major responses (*p* < 0.001). In addition, patients with non-L265P *MYD88* mutations (e.g., S243N, M232T) have been shown to respond favourably to ibrutinib compared to wild-type *MYD88* [5]. *MYD88* mutation status has not been shown to be associated with inferior outcomes when treated with chemoimmunotherapy [19].

### 2.3. Current and Future Treatment Approaches for Patients with WM

Outside the setting of a clinical trial, chemoimmunotherapy remains the treatment of choice in the treatment-naïve patients with LPL. No standard therapy exists. However, rituximab, cyclophosphamide, and dexamethasone (RCD) is highly effective with a 96% ORR and 87% major response rate in treatment naïve WM, and 87% ORR and 68% major response in the relapsed setting. These responses are relatively durable with a 2-year PFS of 67% and median PFS of 34 months in treatment naïve patients [19]. Rummel et al. randomized 41 patients with WM to rituximab in combination with bendamustine (BR) or cyclophosphamide, doxorubicin, vincristine and prednisolone (R-CHOP) and found a significant difference in median progression free survival (PFS; 69.5 versus 28.1 months, *p* = 0.0033) [20]. However, in patients considered unsuitable for these approaches, other treatments are needed.

Ibrutinib has been shown to be highly active in the relapsed setting (2-year PFS 69%, median PFS not reached) [5]. Furthermore, ibrutinib has been shown to be well tolerated with less frequent grade ≥3 neutropenia (14% vs. 20%) and infection (none directly related to ibrutinib vs. 3%) but with a higher risk of grade ≥3 thrombocytopenia (13% vs. 7%), grade ≥2 bleeding (6%) and atrial fibrillation (5%) when compared to chemoimmunotherapy [19,20]. Notably, ibrutinib has not been shown to induce a paraprotein ‘flare’ as is associated with rituximab but does induce a peripheral blood lymphocytosis [18]. A phase 1 study of BGB-3111, a highly-specific irreversible second generation BTK inhibitor with greater selectivity than ibrutinib for BTK has shown significant anti-tumor activity in WM. Tam et al. treated 31 patients with relapsed/refractory and previously untreated WM with BGB-3111 and reported a 92% ORR and 83% major response rate after a median follow-up of 7.6 months (2–21 months) [21]. BGB-3111 was well tolerated with 71% of patients reporting no drug related AE (>grade 1) within the first 12 weeks of therapy and no cases of serious haemorrhage (≥grade 3 or CNS haemorrhage of any grade). Analysis of response by genomic characteristics including *MYD88* and *CXCR4* mutational status are awaited. Patients with heavily pre-treated or chemorefractory disease should be offered therapy with BTK inhibitors when these agents are available.

Venetoclax, a highly selective BCL-2 inhibitor has also been shown to have significant activity in WM. A phase 1 study by Davids et al. [22] identified 106 patients with relapsed/refractory non-Hodgkin’s lymphoma (NHL) treated with venetoclax monotherapy which included four patients with WM. All patients with WM responded (all PR, no CRs) with the duration of response ranging between 11 to 41 months [22]. Treatment was reasonably well tolerated. Grade ≥3 events were uncommon with anaemia (15%), neutropenia (11%), thrombocytopenia (9%) the most frequent and 3% of patients developing laboratory tumour lysis syndrome with no clinical sequelae. Clinical trials investigating the potential synergistic combinations of venetoclax and BTK inhibitors in other subtypes of indolent NHL are currently in progress (NCT02956382, NCT03112174).

## 3. Conclusions

*MYD88* mutation status is useful in the diagnosis, prognostication and prediction of response to targeted therapy in LPL. Further studies of *MYD88* and its downstream signalling pathways are required to better understand mechanisms of disease resistance to BTK inhibitors. Ongoing prospective studies will hopefully determine the role of these highly effective agents in patients with WM, including in previously untreated patients.
